# Crude triterpenoid saponins from *Ilex latifolia* (*Da Ye Dong Qing*) ameliorate lipid accumulation by inhibiting SREBP expression via activation of AMPK in a non-alcoholic fatty liver disease model

**DOI:** 10.1186/s13020-015-0054-9

**Published:** 2015-08-20

**Authors:** Rui-Bing Feng, Chun-Lin Fan, Qing Liu, Zhong Liu, Wei Zhang, Yao-Lan Li, Wei Tang, Ying Wang, Man-Mei Li, Wen-Cai Ye

**Affiliations:** College of Pharmacy, Jinan University, Guangzhou, 510632 People’s Republic of China; Guangdong Province Key Laboratory of Pharmacodynamic Constituents of TCM and New Drugs Research, Jinan University, Guangzhou, 510632 People’s Republic of China; Guangzhou Jinan Biomedicine Research and Development Center, National Engineering Research Center of Genetic Medicine, Jinan University, Guangzhou, 510632 People’s Republic of China

## Abstract

**Background:**

*Ilex latifolia* Thunb. (*Da Ye Dong Qing*) is used for weight loss and for its antidiabetic effects. This study aims to investigate the beneficial effects and potential mechanisms of action of crude triterpenoid saponins (CTS) from *I. latifolia* in a mouse model of high-fat diet (HFD)-induced non-alcoholic fatty liver disease (NAFLD).

**Methods:**

Male C57BL/6 mice (n = 50), were arbitrarily divided into five groups (n = 10 in each group): a control group, HFD group, simvastatin group (10 mg/kg/day), and two CTS treatment groups (100 and 200 mg/kg/day). All mice except those in the control group were fed an HFD for 4 weeks. Animals in the treatment groups were orally administered simvastatin or CTS for 8 weeks. Oral glucose tolerance tests and insulin tolerance tests were performed. At the end of treatment, plasma lipid levels, and oxidative parameters in the liver were measured using commercial test kits. Western blotting was used to evaluate whether CTS induced AMP-activated protein kinase (AMPK) and acetyl CoA carboxylase activation, and the expression of transcription factors and their target genes was evaluated in a quantitative PCR assay.

**Results:**

Compared with the HFD group, the CTS (200 mg/kg/day) treatment group showed significantly decreased plasma lipid parameters (*P* < 0.001, *P* = 0.018, and *P* = 0.005 for triglycerides, total cholesterol and low-density lipoprotein cholesterol, respectively), and improved insulin resistance (*P* = 0.006). CTS (100 and 200 mg/kg/day) supplementation also reduced hepatic lipids and protected the liver from oxidative stress by attenuating malondialdehyde content (*P* < 0.001 and *P* < 0.001, respectively) and restoring aspartate aminotransferase levels (*P* < 0.001 and *P* < 0.001, respectively). Moreover, CTS (200 mg/kg/day) reduced lipid accumulation by enhancing AMPK phosphorylation and inhibiting expression of sterol regulatory element-binding proteins (SREBPs) and their target genes SREBP-1c, SREBP-2, fatty acid synthase, and stearoyl-CoA desaturase (*P* = 0.013, *P* = 0.007, *P* = 0.011, and *P* = 0.014, respectively).

**Conclusion:**

CTS from *I. latifolia* improved insulin resistance and liver injury in HFD-fed mice, and attenuated NAFLD via the activation of AMPK and inhibition of the gene expression of SREBPs and some of their target molecules.

## Background

Non-alcoholic fatty liver disease (NAFLD) is characterized by fatty changes in the liver that do not occur as a result of alcohol intake. It refers to a wide spectrum of conditions involving liver damage, including non-alcoholic steatohepatitis (NASH), fibrosis, and cirrhosis and its complications [1, 2], and is now regarded as a manifestation of metabolic syndrome and a shared pathogenic factor for obesity, type 2 diabetes mellitus (T2DM), and cardiovascular disease [3, 4]. In terms of its pathogenesis, excessive fat accumulation in hepatic cells causes oxidative stress in the liver, leading to the development of insulin resistance (IR) [5, 6]. Thus, reducing fat accumulation by inhibiting lipogenic synthesis and increasing fat breakdown could be helpful in the prevention and treatment of NAFLD.

Many enzymes and transcription factors are involved in lipogenesis and are organized into multi-enzyme complexes to promote fatty acid synthesis, including acetyl CoA carboxylase (ACC), fatty acid synthase (FAS), stearoyl-CoA desaturase 1 (SCD-1), and sterol regulatory element-binding protein (SREBP) [7, 8]. In the liver, SREBP-2 predominantly regulates enzymes associated with cholesterol and bile acid synthesis, such as hydroxymethylglutaryl coenzyme A synthase (HMGCS) [9]. AMP-activated protein kinase (AMPK) is a serine/threonine protein kinase composed of α, β, and γ subunits, and is associated with adipocyte differentiation [10]. Furthermore, increasing evidence indicates an inverse correlation between AMPK and SREBP-1c in hepatocytes and the livers of high-fat diet (HFD)-fed or ethanol-fed mice [11, 12]. AMPK interacted with and phosphorylated SREBP-1c and SREBP-2 to attenuate hepatic steatosis in mice with diet-induced IR [13]. Therefore, the regulation of AMPK and SREBP may be of key therapeutic importance in preventing fatty liver disease.

The leaves of *Ilex latifolia* Thunb. (*Da Ye Dong Qing*) have been widely used to quench thirst, refresh the mind, relieve *wind*-*heat* (*feng*-*re*), and clear toxins from the *blood* (*xue*); they are also used to treat the common cold, obesity, sore throat, rhinitis, itching eyes, and conjunctival congestion [14]. Furthermore, *I. latifolia* may improve hypertension, hyperlipidemia, and other cardiovascular diseases [15, 16]. Phytochemical studies have indicated that triterpenoids, phenolic acids, flavonoids, and essential oils are the major constituents of *I. latifolia* [17–19]. Many of these components have potential health benefits including antioxidant, anti-obesity, neuro-protective, hepatoprotective, and anti-inflammatory effects [20–23]. Triterpenoids from *I. latifolia* were shown to inhibit acyl CoA cholesterol acyl transference [24].

This study aims to investigate the beneficial effects and potential mechanisms of action of crude triterpenoid saponins (CTS) from *I. latifolia* in a mouse model of HFD-induced NAFLD.

## Methods

### Plant material

The leaves of *I. latifolia* were collected from Wanning, Hainan Province, China, and were identified by Prof. Guangxiong Zhou, College of Pharmacy, Jinan University, Guangzhou, China. The voucher specimen (No. 1201209) was deposited at the College of Pharmacy, Jinan University.

### Chemicals and reagents

Kudinoside O, kudinoside N, kudinoside H, latifoloside G, kudinoside G kudinoside C, kudinoside A, kudinoside E, kudinoside F, latifoloside Q, kudinoside D, and ilekudinoside E were extracted from the leaves of *I. latifolia* using boiling water, separated using D101 macroporous resin (Yunkai Resin Technology Co., Ltd., China) and silica gel column chromatography (Qingdao Haiyang Chemical Co., Ltd., China), and purified using high performance liquid chromatography (HPLC) column chromatography (Cosmosil 5C18-MS-II, Nacalai Tesque, Japan) and a preparative HPLC system (Agilent 1260, Agilent Technologies, USA). The structures of these saponins were determined by comparing their spectral (UV, IR, MS, and NMR) data with those in the literature [15, 25–27]. Each saponin was dissolved in methanol at a concentration of 20 μg/mL. HPLC-grade acetonitrile, methanol, and formic acid were purchased from Merck (Darmstadt, Germany). Rabbit polyclonal antibodies against phospho-AMPKα (Thr172), AMPK, phospho-ACC, and ACC were purchased from Cell Signaling Technology (Danvers, MA, USA). All the other chemical reagents were obtained from Sigma-Aldrich (St. Louis, MO, USA). Normal diets and the HFD were purchased from Guangdong Medical Laboratory Animal Center (Guangzhou, China). The HFD consisted of 18% lard stearin (w/w), 5% egg powder, 1% cholesterol, 20% sucrose, 0.1% bile salt, and 55.9% normal diet.

### CTS preparation

The leaves of *I. latifolia* were extracted three times with boiling water for 2 h. The extracting solution was applied to a D 101 resin column and eluted with H_2_O, 45% EtOH, 75% EtOH, and 95% EtOH successively to obtain four fractions. Our previous studies, in which we performed a chemical analysis of the leaves of *I. latifolia* using ultra-high-performance liquid chromatography quadrupole time-of-flight mass spectrometry (UPLC-QTOF–MS), showed the presence of CTS in the 75% EtOH fractions [28]. The 75% EtOH fraction was concentrated and dried in a vacuum to obtain the CTS.

### Determination of CTS content

CTS were qualitatively and quantitatively analyzed by UHPLC-Q-TOF-MS. The CTS (20 mg) were dissolved in 100 mL of methanol, and filtered with a 0.22-μm nylon filter membrane. The samples were prepared in triplicate. The filtrate was then transferred to an auto-sampler vial for UHPLC-Q-TOF-MS analysis. The injection volume was 2 μL.

UHPLC was performed on a Waters Acquity UHPLC system (binary solvent delivery system, auto sampler, and column compartment; Waters, Milford, MA, USA), which was coupled with a Waters Xevo G2 Q-TOF (Waters). The samples were separated on an Agilent Extend C18 RRHD column (2.1 × 150 mm I.D., 1.8 μm). The mobile phase consisted of H_2_O containing 0.1% formic acid (v/v, solvent A) and acetonitrile containing 0.1% formic acid (v/v, solvent B). A gradient program was used according to the following profile: 0–1 min, 5% B; 1–4 min, 5–25% B; 4–12 min, 25–40% B; 12–13 min, 40–95% B; and 3-min post-run, 5% B, flow rate 0.5 mL/min. The column temperature was 40°C. The interface was electrospray injection, and the acquisition parameters were as follows: capillary voltage, 3,500 V; cone voltage, 5 V; source temperature, 100°C; nebulization gas, 800 L/h at 350°C; cone gas, 50 L/h; and mass range recorded, *m*/*z* 100–15,00. During the whole process of analysis, leucine-enkephalin (*m*/*z* 554.2615) served as a reference ion was sampled 0.3 s per 5 s. Quantification was performed using the negative ion mode.

### Animals and their treatment

Six-week-old, male C57BL/6J mice (n = 50, specific pathogen-free grade, Certified No. SCXK [Guangdong] 2003-0001) were obtained from Guangdong Medical Laboratory Animal Center (China). The animals were kept in a breeding room with controlled environment (temperature: 23 ± 1°C, 12 h dark/light cycle from 6:00 a.m. to 6:00 p.m.) with free access to food and water. After a week of adaptation, the mice were arbitrarily divided into five groups (n = 10/group) as follows: a control group (Control group), an HFD-fed group (HFD group), treatment groups fed the HFD for 4 weeks followed by administration of CTS at 100 mg/kg/day or 200 mg/kg/day, or a positive control group treated with simvastatin (10 mg/kg/day) (Laboratories Fournier S.A., USA) by oral gavage for 8 weeks. During the experiment, animal body weight was recorded each week, and the control and HFD groups received equal volumes of saline for 8 weeks. After 8 weeks of treatment, all animals were sacrificed under chloral hydrate anesthesia and blood was collected by cardiac puncture. The main part of the visceral fat was removed from each group and weighed. The livers and blood plasma were collected and stored at −80°C for further biochemical analysis. All animal experiments were conducted in accordance with the guidelines (publication number 85–23, revised 1996) set by the National Institutes of Health and the U.S. Department of Agriculture, and were approved by the local animal ethics committee of Jinan University.

### Oral glucose tolerance test (OGTT)

In a separate trial, glucose tolerance was analyzed as previously described [29]. After 50 days of treatment with the HFD, mice were fasted overnight and orally administered 2 g/kg glucose. Glucose levels were measured in tail blood before and at 30, 60, or 120 min after oral administration using a blood glucose monitoring system (ACCU-CHEK^®^ Active, Roche, Germany). The area under the curve (AUC) of blood glucose and time was calculated according to the following formula:

$$ {\text{AUC}}_{{0{-}2{\text{ h}}}} = \left[ {\left( {{\text{G}}_{{0{\text{ h}}}} + {\text{G}}_{{1{\text{ h}}}} } \right) \times 1 {\text{h}} + \left( {{\text{G}}_{{1{\text{ h}}}} + {\text{G}}_{{2{\text{ h}}}} } \right) \times 1 {\text{h}}} \right]/2 $$

where “G” is the blood glucose value.

### Insulin tolerance test (ITT)

The ITT was performed as previously described [29]. After 60 days of treatment with the HFD, mice were fasted overnight and subcutaneously injected with 0.75 U/kg insulin (Novolin, Novo Nordisk, Denmark). Glucose levels were measured from tail blood before and 15, 30, 60, or 120 min after injection with a blood glucose monitoring system (ACCU-CHEK^®^Active, Roche, Germany). All animals were sacrificed 3 days after the ITT. Plasma insulin levels were measured with the insulin radioimmunoassay kit (North Institute of Biological Technology, China) according to the manufacturer’s instructions.

### Measurement of biochemical parameters in plasma

Plasma was separated by centrifuge (Eppendorf 5417R; Eppendorf, Germany) at 1,699×*g* for 15 min at 4°C. The triglyceride (TG) and total cholesterol (TC) contents in plasma were measured using TG and TC quantification kits (Nanjing Jiancheng Bioengineering Institute, China). Blood glucose, low-density lipoprotein cholesterol (LDL-c), and high-density lipoprotein cholesterol (HDL-c) levels were determined according to the protocols of commercially available kits (Nanjing Jiancheng Bioengineering Institute). The enzymatic activities of plasma aspartate aminotransferase (ALT) and alanine aminotransferase (AST) were estimated for assessing liver damage using biochemical kits (Nanjing Jiancheng Bioengineering Institute).

### Assay of hepatic levels of free fatty acid (FFA), glutathione (GSH), malondialdehyde (MDA), and adenosine triphosphatase (ATPase)

The content of FFA in the liver was measured using a commercially available kit according to the manufacturer’s protocol (Nanjing Jiancheng Bioengineering Institute). Hepatic tissues were homogenized (10%, w/v) in ice-cold saline (pH 7.4) and centrifuged at 1,699×*g* for 10 min at 4°C. The supernatants were transferred to new centrifuge tubes and the MDA, GSH, and ATPase contents were measured using biological test kits. Lipid peroxidation was estimated by measuring thiobarbituric acid-reactive substances (TBARS), and is expressed in terms of MDA content according to the manufacturer’s protocol. The reagent products were pink in color and absorbance was measured using an MK3 microplate reader (Thermo Company, MA, USA) at 532 nm. Protein content was determined using a protein assay kit (Bio-Rad Laboratories Inc., USA) to normalize MDA, GSH, and ATPase levels.

### Oil red O staining of liver

For microscopic analysis of the effect of CTS treatment on hepatic lipid accumulation induced by the HFD, 0.5 g/100 ml Oil red O (Sigma-Aldrich, St Louis, MO, USA) stock solution was prepared in isopropanol. Liver tissues (5 μm) were frozen, embedded in optimal cutting temperature medium, stained with Oil red O for 15 min and washed, and finally, counterstained with hematoxylin. The samples were photographed and examined using a light microscope (Olympus, Japan) at a magnification of 400×.

### Hepatic lipid content analysis

Liver samples from the five groups were weighed and homogenized in tissue lysis buffer containing 20 mM Tris HCl (pH 7.5), 150 mM NaCl, 1% Triton X-100; lipid was extracted with chloroform/methanol (2:1, v/v).The chloroform layers were separated to measure hepatic TG and cholesterol levels using triglyceride and cholesterol kits according to the manufacturer’s instructions.

### RNA extraction and real-time reverse transcription polymerase chain reaction (RT-PCR)

Total RNA was extracted from mouse liver tissues using Trizol reagent (Invitrogen, Carlsbad, CA, USA) following the manufacturer’s directions to determine the effects of CTS on TG- and cholesterol-related gene expression. Reverse transcription (RT) by total RNA (1 μg), oligo (15) dT, and reverse transcriptase was performed using RT kits (Takara Biotechnology, Inc., Shiga, Japan) in a final volume of 50 μL. PCR primer sequences were designed using primer 6.0 software and synthesized by Invitrogen Technologies (China). The gene expression levels were analyzed in duplicate using a SYBR Green kit (Takara Biotechnology, Inc.) on a real-Time PCR System (Light Cycler 480, Roche, Switzerland). All the primer lengths and annealing temperatures are listed in Table 1. The values of the threshold cycle (C_t_) were determined using Roche analysis software (Light Cycler 480). The relative gene expression levels were normalized to the GADPH expression level, and the ratios are presented as arbitrary units.

**Table 1:** Primer sequences used for real-time PCR

Genes	Primer sequences		Length
HMGCS	Forward	(5′-3′) GCCGTGAACTGGGTCGAA	77 bp
	Reverse	(5′-3′) GCATATATAGCAATGTCTCCTGCAA	
ACC	Forward	(5′-3′) TGAAGGGCTACCTCTAATG	182 bp
	Reverse	(5′-3′) TCACAACCCAAGAACCAC	
SCD-1	Forward	(5′-3′) CCGGAGACCCCTTAGATCGA	190 bp
	Reverse	(5′-3′) TAGCCTGTAAAAGATTTCTGCAAACC	
SREBP-2	Forward	(5′-3′)GCGTTCTGGAGACCATGGA	131 bp
	Reverse	(5′-3′) ACAAAGTTGCTCTGAAAACAAATCA	
SREBP-1c	Forward	(5′-3′)TGTTGGCATCCTGCTATCTG	189 bp
	Reverse	(5′-3′) AGGGAAAGCTTTGGGGTCTA	
FAS	Forward	(5′-3′) GCTGCGGAAACTTCAGGAAAT	84 bp
	Reverse	(5′-3′) AGAGACGTGTCACTCCTGGACTT	
GAPDH	Forward	(5′-3′) AGGTCGGTGTGAACGGATTTG	123 bp
	Reverse	(5′-3′) TGTAGACCATGTAGTTGAGGTCA	

### Western blotting analysis

Fresh liver tissues were homogenized and lysed in ice-cold buffer for 30 min, and tissue lysate samples were centrifuged at 15,294×*g* for 20 min at 4°C. The supernatants were collected and the protein concentrations of the lysates were measured using a BCA protein assay (Bio-Rad Laboratories Inc.). Twenty micrograms of total protein was separated by 8% SDS–polyacrylamide gel electrophoresis (SDS-PAGE), electrotransferred to a PVDF membrane (Millipore, MA, USA), blocked, and immunoblotted with rabbit polyclonal antibodies specific for phospho-AMPKα, AMPK, phospho-ACC, and ACC (diluted 1:1,000) overnight at 4°C. The blots were incubated with a goat anti-rabbit IgG HRP-conjugated secondary antibody for 2 h at room temperature after being washed. Antibody binding on the PVDF membrane was detected using an enhanced chemiluminescence western blot detection kit (Invitrogen, USA) and Kodak X-ray film.

### Statistical analysis

Data were analyzed using Origin version 8.0 software (Origin Lab, USA) and are presented as the mean ± standard deviation (SD). Student’s *t* test was used to compare the difference between two groups, and one-way analysis of variance (ANOVA) was used to perform multiple comparisons. The dose-dependent manner was visually determined from the graphs and the tendency of the data. *P* values less than 0.05 were considered statistically significant and a *P* value less than 0.01 was considered very statistically significant; exact P-values are shown unless *P* < 0.001.

## Results

### CTS content in *I. latifolia*

**Fig. 1:**
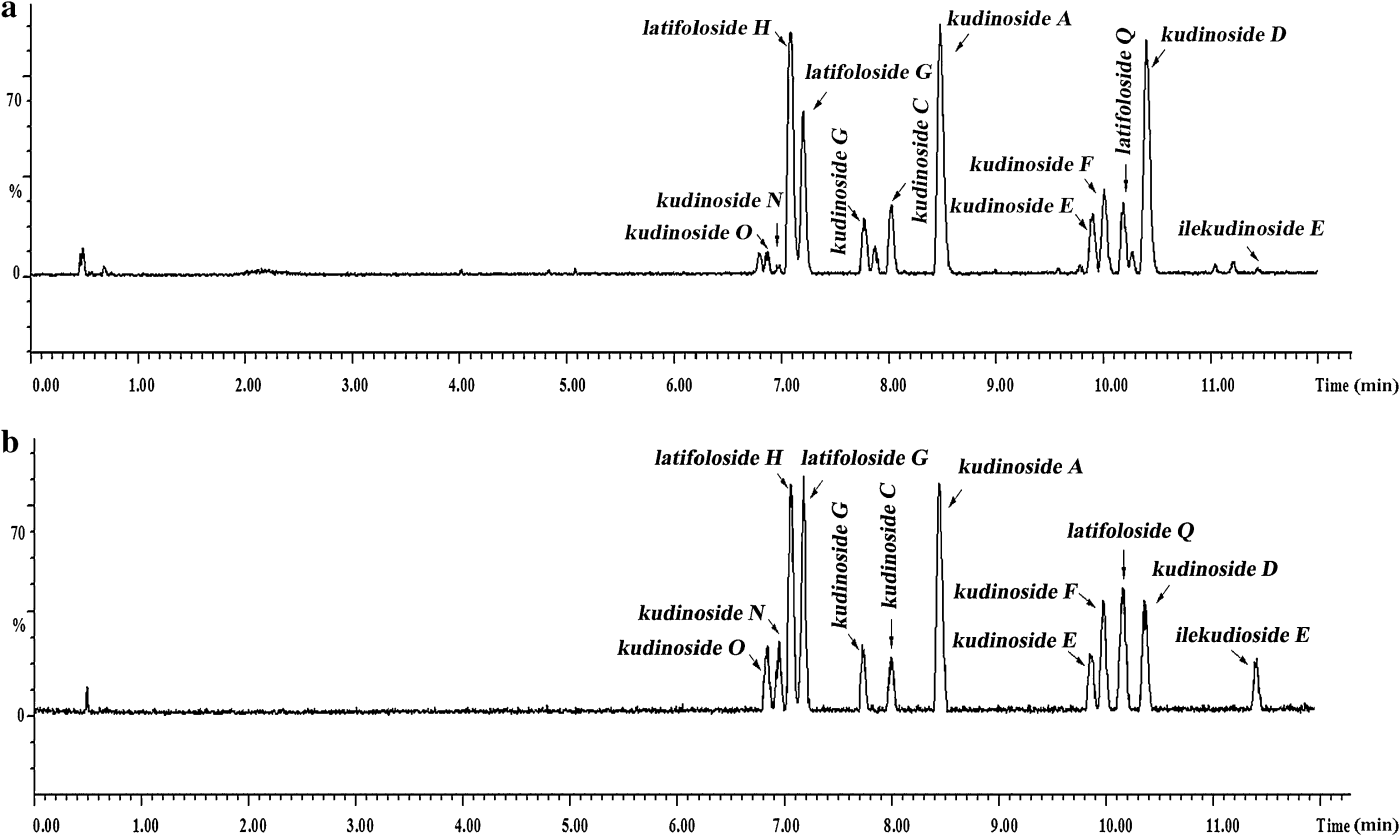
Total ion chromatograms of CTS from *I. latifolia* (**a**) and 12 reference compounds (**b**) detected in negative ion mode.

There were significant differences in the quantities of the 12 main saponins (Fig. 1; Table 2) The contents of the 12 main saponins were obtained directly using an external standard method. The content of kudinoside D (196.68 mg/g) was the highest, followed by those of kudinoside A (117.97 mg/g), latifoloside H (104.17 mg/g), and kudinoside C (95.57 mg/g). The total amount of the twelve main saponins making up CTS was 861.03 mg/g.

**Table 2:** The chemical structures and contents of 12 triterpenoid saponins in the extract of *I. latifolia*

No.	Constituents	Structure							Experimental negative QTOF-MS (m/z)/error (ppm)	Weight of concentration (mg/g)
		Type	R_1_	R_2_	R_3_	R_4_	R_5_	=		
1	Kudinoside O	A	S_4_	OH	H	CH_3_	S_2_	12 (13)	1381.6641 [M − H]^−^/0.31	50.63
2	Kudinoside N	A	S_4_	OH	CH_3_	H	S_2_	12 (13)	1381.6632 [M − H]^−^/0.96	3.45
3	Latifoloside H	A	S_3_	OH	H	CH_3_	S_2_	12 (13)	1265.6165 [M + COOH]^−^/0.56	104.17
4	Latifoloside G	A	S_3_	OH	CH_3_	H	S_2_	12 (13)	1265.6173 [M + COOH]^−^/−0.09	45.76
5	Kudinoside G	A	S_3_	OH	CH_3_	H	S_1_	12 (13)	1119.5595 [M + COOH]^−^/−0.21	65.84
6	Kudinoside C	B	S_4_	H	OH	–	–	13 (18)	1133.5375 [M + COOH]^−^/0.96	95.57
7	Kudinoside A	B	S_3_	H	OH	–	–	13 (18)	971.4853 [M + COOH]^−^/0.45	117.97
8	Kudinoside E	B	S_4_	H	H	–	–	11 (12), 13 (18)	1115.5287 [M + COOH]^−^/−0.68	78.31
9	Kudinoside F	B	S_3_	OH	H	–	–	13 (18)	971.4848 [M + COOH]^−^/0.88	61.16
10	Latifoloside Q	A	S_3_	H	H	CH_3_	S_2_	12 (13)	1249.6226 [M + COOH]^−^/−0.27	33.99
11	Kudinoside D	B	S_3_	H	H	–	–	11 (12), 13 (18)	953.4759 [M + COOH]^−^/−0.82	196.68
12	Ilekudinoside G	B	S_4_	H	H	–	–	12 (13)	1117.5430 [M + COOH]^−^/0.58	7.50
Crude triterpenoid saponins										861.03

### CTS reduce the body, liver, and adipose tissue weights in mice fed the HFD

Compared with the control group, mice fed the HFD for 12 weeks exhibited an obvious increase in body, liver, and adipose tissue (omental adipose tissue, perirenal adipose tissue, epididymal adipose tissue) weights (*P* = 0.007, *P* < 0.001, and *P* < 0.001, respectively). A significantly lower weight gain was observed in the groups treated with CTS-100 mg/kg/day (*P* = 0.037) or CTS-200 mg/kg/day (*P* = 0.022) than in the HFD group. Adipose tissue weight was slightly lower in the CTS-100 mg/kg/day group (*P* = 0.050) than in the HFD group. The liver weight and the ratios of liver to body weight in the HFD group were markedly higher than those in the other groups (*P* < 0.001 and *P* < 0.001, respectively). Compared with the HFD group, the CTS-200 mg/kg/day group showed significantly decreased weight of adipose tissue and ratio of adipose tissue to body weight (*P* = 0.027 and *P* = 0.014, respectively). The HFD induced hepatic steatosis and predominantly increased liver, adipose tissue, and body weights. Treatment with CTS inhibited the progression of hepatic steatosis and weight gain in the liver and adipose tissue (Table 3).

**Table 3:** Effects of CTS on body weight, liver weight, and adipose tissue weight of mice fed with HFD

Parameter	Control	HFD	HFD + Simvastatin	HFD + CTS-100	HFD + CTS-200
Initial body weight (g)	21.10 ± 1.62	21.22 ± 1.21	20.31 ± 1.03	21.56 ± 2.02	21.64 ± 1.36
Final body weight (g)	30.35 ± 3.06	40.39 ± 3.02^##^	35.63 ± 4.63^△^	37.97 ± 4.13*	37.68 ± 2.91*
Body weight gain (g)	9.25 ± 0.91	19.17 ± 0.32^#^	15.32 ± 0.53^△^	16.41 ± 0.44*	14.89 ± 0.46*
Adipose tissue^a^ weight (g)	0.96 ± 0.23	1.67 ± 0.49^##^	1.24 ± 0.29^△^	1.37 ± 0.64	1.43 ± 0.54*
Ratio of adipose tissue and body weight (%)	3.22 ± 0.90	4.29 ± 1.55^##^	3.51 ± 1.49^△^	3.67 ± 2.16	3.84 ± 1.46*
Liver weight (g)	1.45 ± 0.14	2.26 ± 0.17^##^	2.02 ± 0.39^△△^	2.15 ± 0.22	2.09 ± 0.39**
Ratio of liver and body weight (%)	4.81 ± 0.60	5.74 ± 0.71^##^	5.50 ± 1.47^△^	5.70 ± 0.70	5.63 ± 1.30*

Adipose tissue^a^: including omental adipose tissue, perirenal adipose tissue, and epididymal adipose tissue.

Values were expressed as the mean ± SD (n = 10). ^#^ *P* < 0.05, ^##^ *P* < 0.01, HFD vs. control. ^△^ *P* < 0.05, ^△△^ *P* < 0.01, HFD vs. simvastatin treatment. *P < 0.05, **P < 0.01, HFD vs. CTS treatment.

### CTS improve plasma lipid parameters in mice fed the HFD

Lipid levels in the blood were measured as shown in Table 4. The plasma TG, TC, and LDL-c levels were markedly higher in the HFD group (1.98 ± 0.54, 6.27 ± 1.38, and 2.80 ± 0.77 μmol/mL, respectively) compared with the control group (1.37 ± 0.46, 2.48 ± 0.23, and 0.32 ± 0.19 μmol/mL) (*P* = 0.050, *P* < 0.001, and *P* < 0.001, respectively). The plasma concentrations of TG, TC, and LDL-c were lower in the simvastatin-treated group and the CTS-administered groups compared with the HFD group, and the levels of TG, TC, and LDL-c in mice treated with CTS-200 mg/kg/day were significantly lower (*P* < 0.001, *P* = 0.018, and *P* = 0.005, respectively). In contrast, CTS-200 mg/kg/d increased the content of HDL-c, similar to simvastatin, whereas the HFD decreased it (*P* = 0.006). Our results suggested that CTS treatment could reduce fat accumulation in HFD-fed C57BL/6 mice.

**Table 4:** Effects of CTS on plasma parameters in mice fed with HFD

Parameter	Control	HFD	HFD + Simvastatin	HFD + CTS-100	HFD + CTS-200
TG (mmol/L)	1.37 ± 0.46	1.98 ± 0.54^#^	1.04 ± 0.26^△△^	1.29 ± 0.34**	1.21 ± 0.15**
TC (mmol/dL)	2.48 ± 0.23	6.27 ± 1.38^##^	5.27 ± 0.20^△^	5.79 ± 0.60	5.59 ± 0.70*
LDL-C (mmol/dL)	0.32 ± 0.19	2.80 ± 0.77^##^	1.95 ± 0.45^△^	2.53 ± 0.28	2.09 ± 0.39**
HDL-c (mmol/dL)	1.33 ± 0.46	0.95 ± 0.10^#^	1.77 ± 0.66^△^	1.25 ± 0.30**	1.36 ± 0.08**
Glucose (mmol/L)	8.61 ± 1.34	9.99 ± 1.56^##^	8.76 ± 1.43^△^	9.80 ± 1.53	9.60 ± 1.21
Insulin (nmol/L)	22.63 ± 5.93	36.56 ± 7.77^##^	25.53 ± 7.03^△^	26.43 ± 9.71*	24.52 ± 6.20**

Values were expressed as the mean ± SD (n = 10). ^#^ *P* < 0.05, ^##^ *P* < 0.01, HFD *vs*. control. ^△^ *P* < 0.05,^△△^ *P* < 0.01, HFD vs. simvastatin treatment. *P < 0.05, **P < 0.01, HFD vs. CTS treatment.

### CTS improve IR in mice

**Fig. 2:**
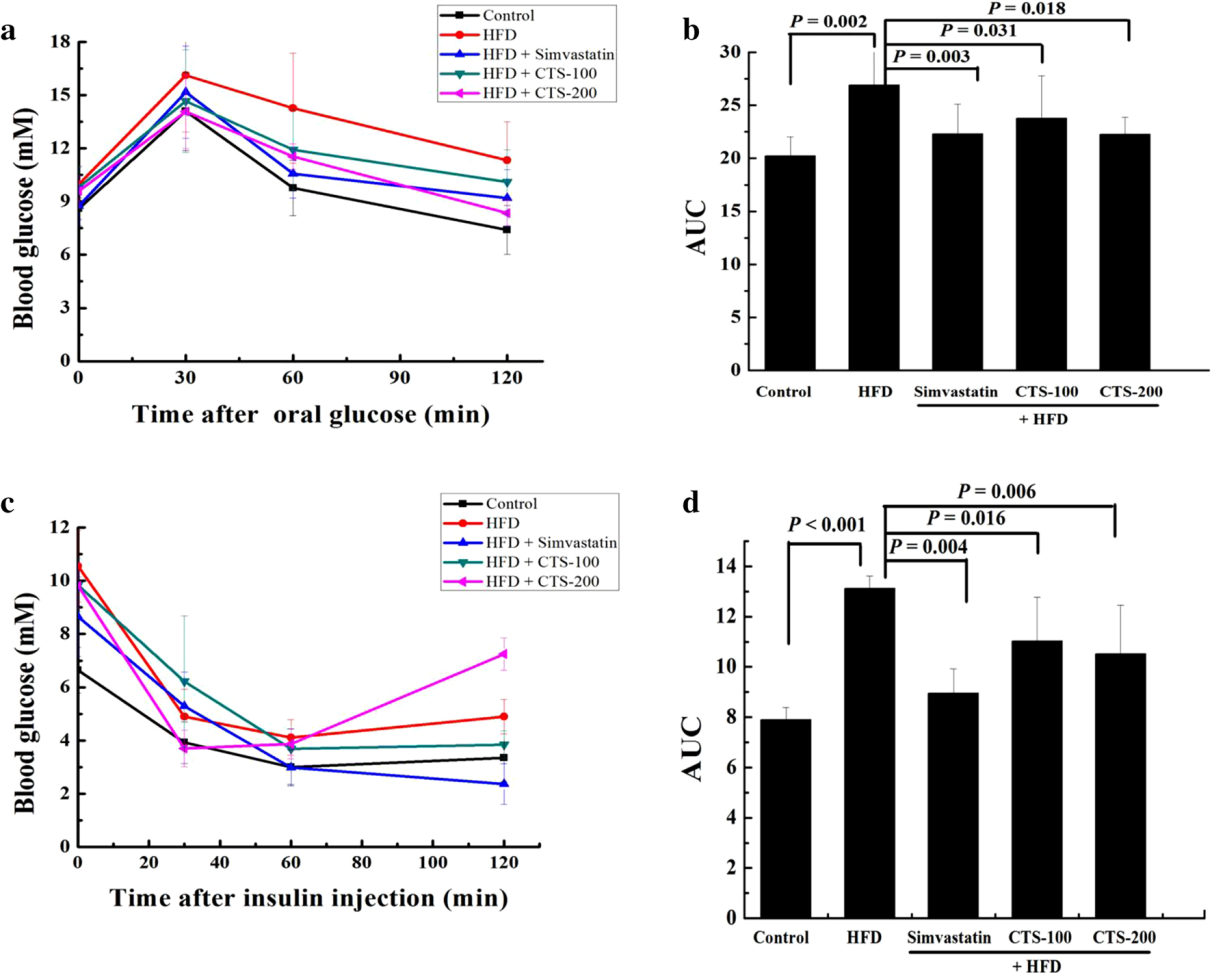
Effects of CTS on the oral glucose tolerance test (GTT) and insulin tolerance test (ITT). The high-fat diet (HFD)-fed mice were fasted overnight and treated with oral intragastric administration of 2 g/kg glucose or a subcutaneous injection of 0.75 U/kg insulin. Simvastatin and CTS reduced the glucose curve induced by glucose and insulin, leading to the eventual improvement in glucose tolerance and insulin resistance. **a** Effects of CTS on glucose tolerance in blood. **b** Effects of CTS on insulin tolerance in blood. **c**, **d** Quantitative analysis of the area under the curve (AUC) from the GTT and ITT. The results are represented as the mean ± SD (n = 10).

After 50 days of CTS treatment, the untreated HFD-fed mice showed a significant increase in blood glucose levels compared with the levels observed in normal controls (Fig. 2a). Simvastatin (10 mg/kg/day) and CTS (100 and 200 mg/kg/day) inhibited this increase in blood glucose levels, and decreased the area under the curve (AUC) of blood glucose over time compared with the AUC for the HFD group (*P* = 0.003, *P* = 0.031, and *P* = 0.018, respectively) (Fig. 2b). Compared with the control group, the HFD-fed mice exhibited impaired insulin tolerance (*P* < 0.001). Simvastatin or CTS-200 mg/kg/day improved insulin tolerance (*P* = 0.004 and *P* = 0.006, respectively) (Fig. 2c, d). Furthermore, CTS-200 mg/kg/day significantly reduced the elevated fasting blood insulin level in HFD-fed mice (*P* < 0.001), while fasting blood glucose was not affected by CTS-200 mg/kg/d (*P* = 0.412) (Table 4). Hepatic FFA content and Ca^2+^-Mg^2+^-ATPase activity were significantly increased in the HFD-fed mice compared with those in normal controls (*P* < 0.001 and *P* = 0.048, respectively). Further, the FFA content and ATPase activity in the liver were highly significantly lower in mice treated with CTS-200 mg/kg/day compared with HFD-fed mice (*P* < 0.001 and *P* = 0.001, respectively) (Table 5).

**Table 5:** Effects of CTS on FFA, MDA, GSH and ATPase in the livers of mice fed with HFD

Parameter	Control	HFD	HFD + Simvastatin	HFD + CTS-100	HFD + CTS-200
FFA (μmol/g protein)	61.05 ± 12.49	94.70 ± 15.63^##^	91.73 ± 13.50^△^	93.89 ± 10.81*	79.87 ± 11.18**
MDA (nmol/mg protein)	0.69 ± 0.34	3.19 ± 0.33^##^	1.88 ± 0.44^△△^	1.68 ± 0.54**	1.50 ± 0.38**
GSH (μg/mg protein)	2.65 ± 0.53	1.05 ± 0.24^##^	2.80 ± 0.59^△△^	2.55 ± 0.65**	2.61 ± 0.50**
Na^+^-K^+^-ATPase (mmol pi/g/h)	1.55 ± 0.19	1.79 ± 018^#^	1.57 ± 0.26^△^	1.68 ± 0.10	1.60 ± 0.26*
Ca^2+^-Mg^2+^-ATPase (mmol pi/g/h)	1.65 ± 0.13	1.76 ± 0.11^#^	1.31 ± 0.14^△^	1.55 ± 0.14**	1.42 ± 0.31**

Values were expressed as the mean ± SD (n = 10). ^#^ *P* < 0.05, ^##^ *P* < 0.01, HFD vs. control. ^△^ *P* < 0.05, ^△△^ *P* < 0.01, HFD vs. simvastatin treatment. *P < 0.05, **P < 0.01, HFD vs. CTS treatment.

### CTS protect mouse liver against oxidative stress-mediated injury induced by lipid overload

**Fig. 3:**
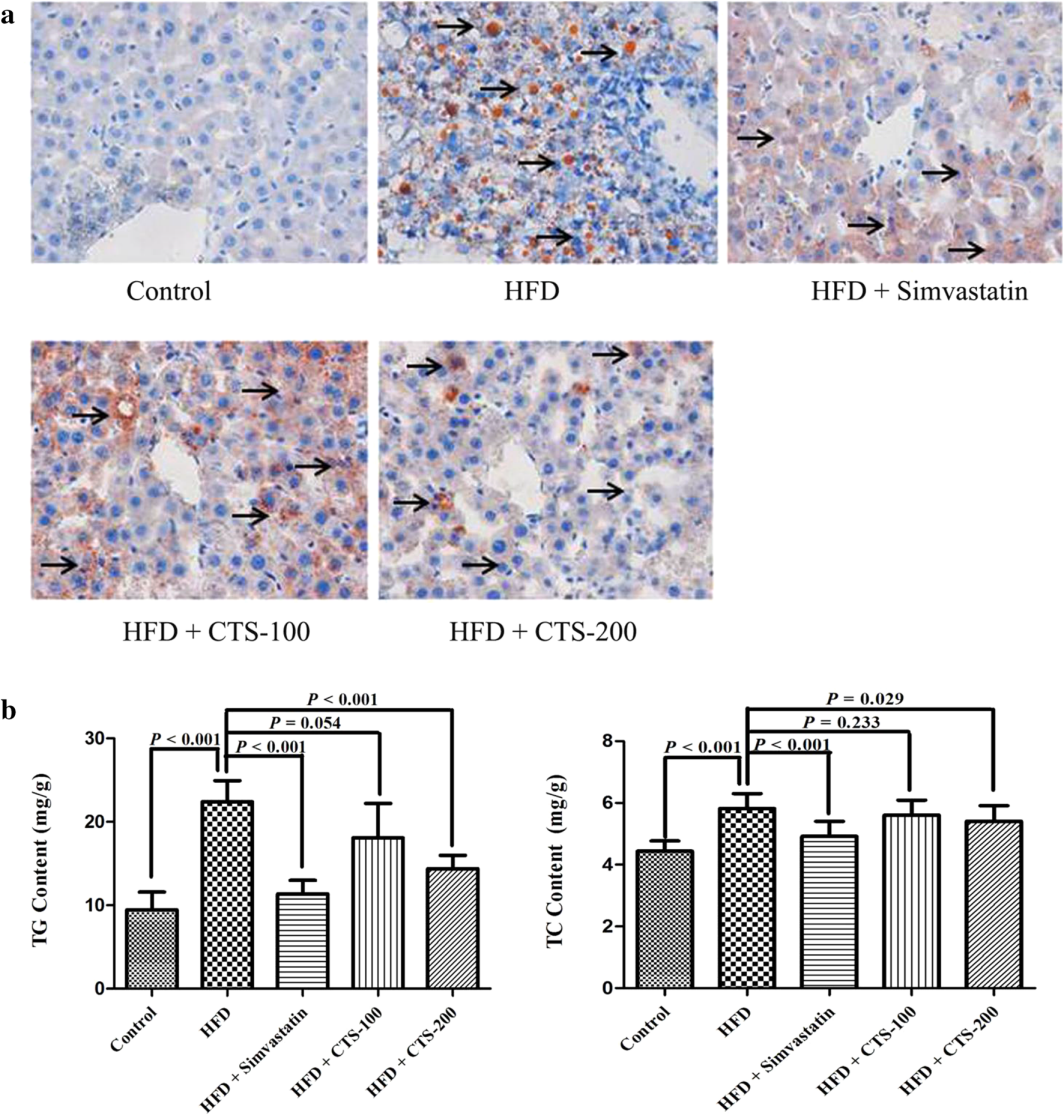
Effects of CTS on liver injury and hepatic lipid content in HFD-fed mice. **a** Oil red O staining (×400) of the liver sections. Arrows indicate steatosis induced by HFD feeding. **b** The quantitative analysis of triglyceride (TG) and total cholesterol (TC) contents in livers. The HFD-fed mice were administered two concentrations of CTS (100 and 200 mg/kg/days) or the positive control simvastatin 10 mg/kg/d for 8 weeks. The results are represented as the mean ± SD (n = 10).

The hepatic TBARS content was determined to evaluate lipid peroxidation levels in the liver and the anti-oxidative status (Table 5). Both results showed that CTS attenuated oxidative stress in the liver. The level of MDA in the liver was four times higher in the HFD group than in the untreated group (*P* < 0.001), and CTS-100 mg/kg/d markedly reduced the level of MDA (from 3.19 ± 0.33 to 1.68 ± 0.54). The level of GSH in the liver was lower in the HFD group than in the control group (*P* < 0.001). After 8 weeks of treatment, CTS (100 and 200 mg/kg/day) significantly increased the GSH level in the liver to a level comparable to that in the control group (*P* < 0.001 and *P* < 0.001, respectively). Plasma ALT and AST activities in different groups were examined to determine whether treatment with CTS could improve the HFD-induced liver injury. The activity of plasma ALT in the HFD group was significantly higher than that in the control group, and both simvastatin and CTS-200 mg/kg/d decreased ALT activity (*P* < 0.001 and *P* < 0.001, respectively). However, there was no significant difference in the level of plasma AST among these groups, except for that in the simvastatin group (Table 6). Hepatic lipids were measured by Oil O red staining and TG content analysis. HFD induced the formation of lipid droplets, which was ameliorated by simvastatin and CTS treatments (Fig. 3). These results suggested that CTS treatment dose-dependently protected against liver damage by suppressing hepatic fat accumulation.

**Table 6:** Effects of CTS on plasma ALT and AST activities in mice fed with HFD

Parameter	Control	HFD	HFD + Simvastatin	HFD + CTS-100	HFD + CTS-200
ALT (KAIU/L)	43.15 ± 3.98	62.61 ± 14.81^##^	51.34 ± 15.81^△△^	50.94 ± 15.95**	40.74 ± 7.90**
AST (KAIU/L)	104.85 ± 26.20	101.74 ± 22.86	127.65 ± 11.83^△△^	96.24 ± 10.18	98.87 ± 12.61

Values were expressed as the mean ± SD (n = 10). ^#^ *P* < 0.05, ^##^ *P* < 0.01, HFD vs. control. ^△^ *P* < 0.05,^△△^ *P* < 0.01, HFD vs. simvastatin treatment. *P < 0.05, **P < 0.01, HFD vs. CTS treatment.

### CTS activate AMPK phosphorylation and inhibit mRNA expression of SREBPs and their target genes

**Fig. 4:**
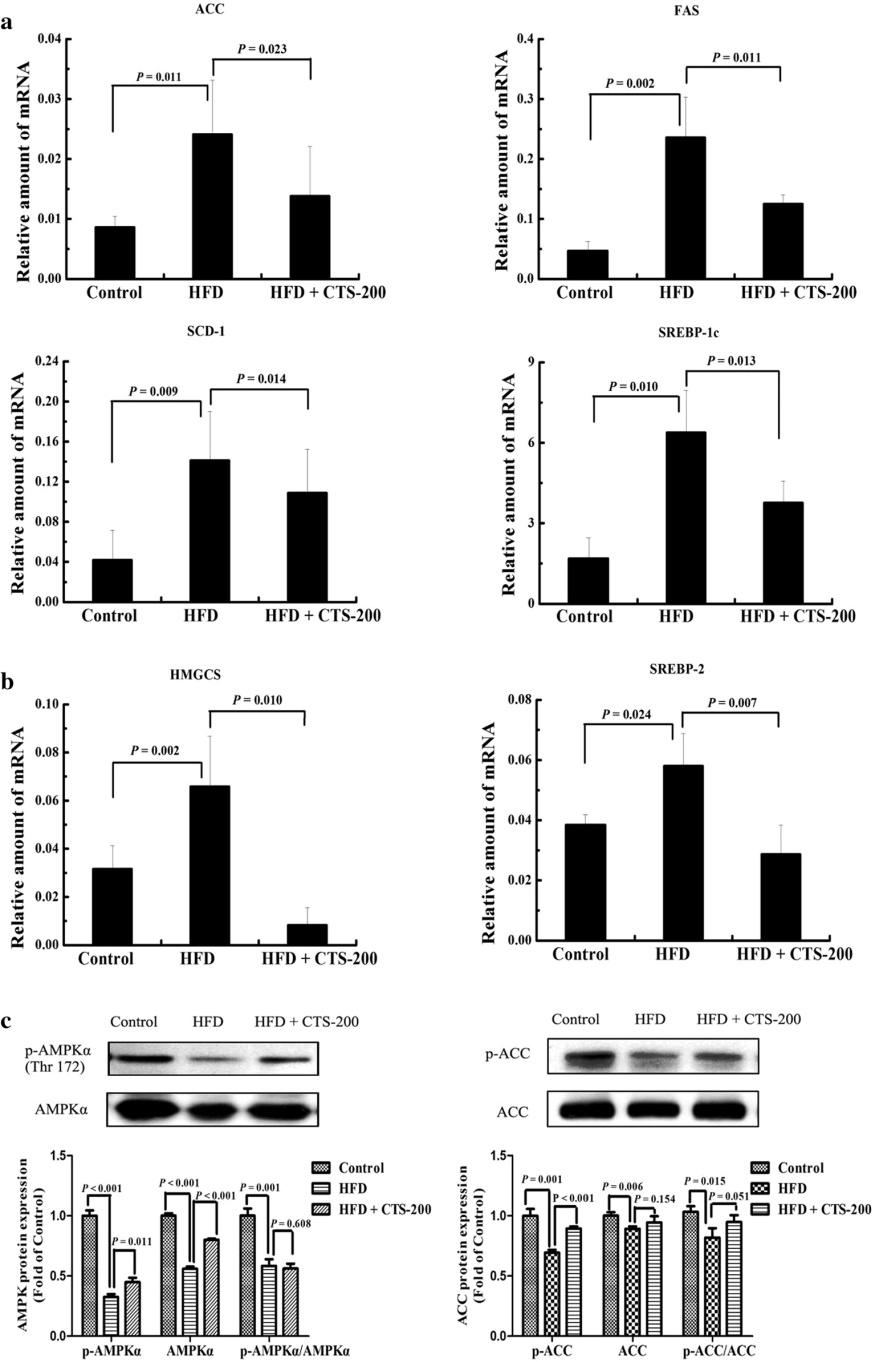
Effects of CTS on gene and protein expression of lipogenic and cholesterol-related synthetic enzymes. **a** The gene expression of lipogenic enzymes (SREBP-1c SCD1, ACC, FAS) in the livers of mice was suppressed by CTS-200 mg/kg/days. **b** The gene expression of cholesterol related synthetic enzymes (SREBP-2, HMGCS) in the livers of mice was suppressed by CTS-200 mg/kg/day. **c** Immunoblot analysis of phospho-AMPKα (Thr172), AMPK, phospho-ACC, and ACC protein. The protein levels are expressed as fold of control. All data were expressed as the mean ± SD (n = 10) of three independent experiments.

HFD-fed mice showed inhibition of AMPKα (Thr172) and ACC protein phosphorylation in the liver (*P* < 0.001 and *P* = 0.001, respectively), but AMPKα and ACC phosphorylation was restored after treatment with CTS-200 mg/kg/day for 8 weeks (*P* = 0.011 and *P* < 0.001, respectively). On the other hand, the mRNA levels of SREBP-1c, FAS, SCD-1, and ACC were significantly inhibited in the CTS-treated group (*P* = 0.013, *P* = 0.011, *P* = 0.014, and *P* = 0.023, respectively). Meanwhile, the expression of the genes in the cholesterol synthetic pathway, such as SREBP-2 and HMGCS, was significantly attenuated in the liver in comparison to that in the HFD-fed group (*P* = 0.007 and *P* = 0.010, respectively) (Fig. 4). Activation of the AMPK-mediated SREBP signaling pathway by CTS might be a potential mechanism underlying the suppression of hepatic fat accumulation.

## Discussion

We showed that orally administered CTS (100 and 200 mg/kg/day) from *I. latifolia* could alleviate NAFLD in HFD-fed mice, and elucidated its underlying mechanisms of suppression of fat accumulation via AMPK pathway activation, to reduce body weight gain, hyperlipidemia, and IR in HFD-fed mice. The depletion of glutathione levels and lipid peroxidation in HFD-fed mice were inhibited by administration of CTS. ALT activity and histologic evidence in the liver showed that CTS treatment prevented liver injury caused by lipid accumulation. CTS could activate AMPK and suppress expression of SREBPs and their target genes.

In previous studies *I. latifolia* was shown to exhibit anti-inflammatory and antioxidant effects and improve lipid metabolism [30, 31]. The water extract of *I. latifolia* inhibited hyperglycemia in rats with epinephrine-induced hyperglycemia [32], and increased the contractility and decreased the frequency of contraction in an isolated rat heart perfusion system by inhibiting Na^+^/K^+^-ATPase activities [33]. Triterpenoid saponins such as ilekudinoside N and ilekudinoside Q from the aqueous extract of *I. kudingcha* reduced intracellular TC and TG contents in macrophages [34]. In addition, the CTS from the aqueous extract could significantly improve metabolic disorders in NAFLD mice [35].

In this study, an HFD-induced NAFLD mouse model was established according to previous research [29]. The HFD-fed group had higher body weight, fat accumulation in liver, and plasma levels of glucose and insulin compared with those in mice fed normal diets. Additionally, HFD caused a lipid metabolism disorder and disrupted the homeostasis of the liver oxidative state, which is an important characteristic in the development of NAFLD. Oral administration of CTS strongly prevented the development of NAFLD by significantly decreasing body and adipose tissue weights. Lipid accumulation in the liver frequently promoted the release of MDA, which diffused into other sites and favored oxidative stress [36, 37]. The increase in MDA content and decrease in GSH level could contribute to apoptosis and nuclear-mitochondrial DNA damage in NASH by producing reactive oxygen species (ROS) [37]. Our results demonstrated that CTS administration could reduce MDA content and restore GSH levels in the livers of HFD-fed mice. Plasma ALT levels indicated a protective effect of CTS on the liver.

IR is involved in the pathological process of NAFLD [38, 39]. Adipose tissue is insensitive to the anti-lipolytic effects of insulin in IR conditions, and the influx of exogenous FFA to the liver is increased. Circulating FFAs promote the production of intracellular metabolites (diacylglycerol), and produce dysfunctional cellular glucose [40]. In contrast, other studies have reported that IR and hyperinsulinemia are linked to the impairment of adenosine triphosphate (ATP) homeostasis [41, 42]. In the present study, the levels of insulin in HFD-fed mice were reduced significantly by CTS administration. Furthermore, CTS could improve HFD-induced glucose and IR. Moreover, CTS significantly reduced the levels of hepatic FFA and the activities of Na^+^-K^+^-ATPase and Ca^2+^-Mg^2+^-ATPase. These results suggested that CTS prevented pathological development of NAFLD by improving IR.

AMPK, an important energy sensor for cellular energy homeostasis, is recognized as a potential therapeutic target for the prevention and treatment of metabolic syndrome [43, 44]. The activation of AMPK in the liver could not only inhibit lipogenesis and gluconeogenesis but also stimulate fatty acid oxidation [45]. Natural products and drugs, such as resveratrol and metformin, could inhibit lipogenesis and eliminate hepatic steatosis in HFD-induced fatty liver by activating AMPK [46, 47]. Activated AMPK could bind phosphorylated SREBP-1c and SREBP-2, negatively regulating their proteolytic processing and elevating the expression levels of their target genes (*ACC*, *FAS*, *SCD1*, and *HMGCS*) in the livers of IR mice [42, 48].The phosphorylation of AMPKα was activated by CTS treatment and the mRNA expression levels of SREBP-1c, FAS, SCD1, and ACC were reduced in the CTS-treated group. Meanwhile, CTS also suppressed the mRNA expression of SREBP2 and HMGCR, which are responsible for cholesterol biosynthesis [49]. CTS inhibited lipogenic synthesis and improved IR in HFD-fed mice by upregulating AMPK activation and suppressing the transcription of SREBPs and their target genes.

## Conclusion

CTS from *I. latifolia* improved IR and liver injury in HFD-fed mice, and attenuated NAFLD via the activation of AMPK and inhibition of the gene expression of SREBPs and some of their target molecules.

## Abbreviations

CTS: crude triterpenoid saponins
HFD: high-fat diet
NAFLD: non-alcoholic fatty liver disease
AMPK: AMP-activated protein kinase
NASH: non-alcoholic steatohepatitis
T2DM: type 2 diabetes mellitus
UHPLC-Q-TOF–MS: ultra-high performance liquid chromatography quadrupole time-of-flight tandem mass spectrometry
MCAO: middle cerebral artery occlusion
TBARS: thiobarbituric acid-reactive substances
RT-PCR: real-time reverse transcription polymerase chain reaction
AUC: area under the curve
SDS-PAGE: SDS-polyacrylamide gel electrophoresis
TG: triglycerides
ACC-1: CoA carboxylase-1
FAS: fatty acid synthase
SCD-1: stearoyl-CoA desaturase
SREBP-1c: sterol regulatory element-binding protein-1c
SREBP-2: sterol regulatory element-binding protein 2
HMGCS: hydroxymethylglutaryl coenzyme A synthase
OGTT: oral glucose tolerance test
ITT: insulin tolerance test
TC: Total cholesterol
LDL-c: low-density lipoprotein cholesterol
HDL-c: high-density lipoprotein cholesterol
ALT: aspartate aminotransferase
AST: alanine aminotransferase
FFA: free fatty acid
GSH: glutathione
MDA: malondialdehyde
ATPase: adenosine triphosphatase
ROS: reactive oxygen species
IR: insulin resistance
